# Editorial: Interferons and GvHD

**DOI:** 10.3389/fimmu.2022.853567

**Published:** 2022-02-03

**Authors:** Boram Kim, Jaebok Choi

**Affiliations:** Division of Oncology, Department of Medicine, Washington University School of Medicine, St. Louis, MO, United States

**Keywords:** interferons, GvHD, allogeneic hematopoietic cell transplant (alloHCT), JAK - STAT signaling pathway, JAK inhibitors, LYG1, CD27, CD70

Allogeneic hematopoietic stem cell transplantation (allo-HSCT) is the most effective treatment and curative approach for patients with hematological malignancies. The life-saving benefits of all-HSCT include graft-versus-leukemia (GvL) effects and healthy immune reconstitution. However, the majority of patients develops graft-vs-host-disease (GvHD), leading to organ/tissue damages in the skin, liver, and GI tract. For that reason, extensive researches on GvHD have been made and identified IFNs as therapeutic targets. Nonetheless, the pleiotropic nature of IFNs has hindered our goals to optimally control GvHD.

The goals of the articles collected in the Research Topic are to define the roles and mechanisms of IFNs in GvHD and to provide insights into novel therapeutic approaches to restrain GvHD while enhancing the benefits of allo-HSCT.

To expand the investigation on mechanisms of primary human T cell responses in GvHD, xenogeneic cell transplant models have been utilized. In depth, Hess et al. highlighted the development of humanized mouse models and xenogeneic transplant model system, leading to investigation on human T cell biology. The models essentially benefit from the human T cell receptors (TCRs) having cross-reactivity to murine MHC in addition to cytokines and co-stimulatory proteins. Hess et al. further explained the importance of TCR, the ability to recognize non-self-antigens on MHC, and cellular therapy by suppressing or enhancing the secretion of cytokines and co-stimulatory/inhibitory signaling pathways.

In the review by Zhao et al. the authors discussed the effects of IFNs on T cell and their function and epigenetic regulations during GvHD. They elaborated the paradoxical roles of IFNs in alloreactive T cell differentiation and function and the development of GvHD. The process of GvHD is also critically controlled by epigenetic regulators, such as DNA methylation, histone modification, and chromatin remodeling. In turn, these regulators modulate T cell differentiation and function by influencing IFN signaling and therapeutic potentials. Conclusively, the authors suggest future studies on epigenetic mechanisms of IFNs to better understand how IFNs regulate GvHD.

Not only essential for differentiation and expansion of immune cells, IFNs play an important role in the GI tract. Haring et al. explored the pathophysiology of GvHD caused by the imbalance of cytokine network in the GI tract. The authors summarize that similar to IBD, type I and type III IFNs are important for maintaining the intestinal epithelial barrier integrity and controlling immune responses in GvHD. However, the conditioning regimen prior to allo-HSCT damages the epithelial cells and results in the release of DAMPs and PAMPs, leading to local inflammation together with inflammatory cytokines and activation of APCs. This in turn results in the activation and expansion of the alloreactive T cells causing further tissue destruction and inflammation together with cytokines including type II IFN and chemokines in the GI tract. Overall, the review shows the highly complex and interconnected cytokines network and the effect of its imbalance on intestinal mucosal inflammation, suggesting that identifying targets downstream of IFNs and receptors might provide novel therapeutic strategies for GvHD and IBD.

The use of JAK inhibitors is a promising therapeutic strategy for GvHD and cytokine release syndrome. Assal and Mapara comprehensively reviewed the role of the JAK/STAT pathway in T cell activation and expansion, APC function, and Tregs expansion. The knowledge led to the idea of disrupting the JAK/STAT pathway by using ruxolitinib or baricitinib to ameliorate GvHD. Clinical evidence has also shown that ruxolitinib administration to GvHD patients has been successful. The study by Wang et al. also showed similar results; in the retrospective study, 70 Chinese patients with steroid-refractory chronic GvHD (SR-cGvHD) received ruxolitinib, then the overall response, complete remission, and partial remission rates were examined. Twenty-four weeks after ruxolitinib treatment, those rates were significantly improved, compared to those of the patient group without ruxolitinib treatment. The authors also showed that CD4^+^ cells, total B cells, and IL-10 were significantly increased after the treatment, whereas NK cells, Tregs, and ST2 were significantly decreased. Collectively, these results suggest that ruxolitinib is a safe and effective treatment for SR-cGvHD.

Targeting TCR and co-stimulatory pathways also serves as a therapeutic strategy to control GvHD. In the review by Lutfi et al. authors illustrated the CD27-CD70 co-stimulatory pathway and its therapeutic potential in combination with immune checkpoint inhibitors and in GvHD. The CD27-CD70 pathway leads to survival and activation of T, B, and NK cells, and the activity is shown to be increased under pro-inflammatory conditions and IFN-γ secretion. With the understanding of its nature, studies to targeting the CD27-CD70 pathway to improve outcomes have been made. For instance, a CD27 agonizing monoclonal antibody, varlilumab, showed to improve antineoplastic response in combination with immune checkpoint inhibitors by enhancing CD8^+^ T cell expansion and proliferation. On the other hand, an administration of anti-CD70 monoclonal antibody and CD70 KO mice resulted in increased GvHD. Further studies explained that while APC-expressed CD70 provides a co-stimulatory signal, T-cell-expressed CD70 served an inhibitory role in T-cell response. These findings proposed that this complex mechanism may provide a potential therapeutic intervention as an oncologic therapy and to attenuate GvHD by modulating the pathway.

Previous studies suggest that recombinant human LYG1 protein (rhLYG1) can promote the activation and IFN-γ production of CD4^+^ T cells, thereby inhibiting tumor growth. Liu et al. in turn hypothesized that LYG1 participated in the development of GvHD and explored the role and mechanisms of LYG1 during GvHD. As hypothesized, the study discovered that LYG1 deficiency in donor T cells reduced the severity of GvHD. In depth, LYG1 deficiency in donor T cells inhibited the activation of CD4^+^ T cells and expression of IFN-γ, while promoting the expression of FOXP3, thereby suppressing the expression of CXCL9 and CXCL10 and the infiltration of allogeneic CD4^+^ T cells into target organs. Despite of a modest improvement in the overall survival by LYG1 blockade, these findings demonstrate that targeting LYG1 may be as a potential therapeutic strategy to reduce GvHD.

Huisman et al. provide significant insight on the risk of off-target toxicity *via* allo-HLA across-reactivity. Using third-party donor-derived virus-specific T cells, Huisman et al. investigated whether HLA-restriction, HLA background, and/or virus-specificity could predict the risk of allo-HLA cross-reactivity. The results demonstrated that HLA-B*08:01-restricted T cells, had the highest allo-HLA cross-reactivity regardless of virus-specificity, suggesting a potential strategy to reduce the risk of off-target toxicity by selecting T cells with a specific HLA restriction and background.

Overall, the articles in the Research Topic provided sufficient reviews on the recent advances in the roles of IFNs in GvHD. They also highlighted promising therapeutic strategies, such as CD27 agonizing monoclonal antibodies, JAK inhibitors, and targeting LYG1, to modulate IFN signaling to restrain GvHD while preserving the benefits of all-HSCT. Indeed, further researches should be followed to reveal the exact mechanisms of IFNs in the context of transplantation to provide promising transplantation therapies.

## Author Contributions

BK and JC wrote the manuscript. JC is a guest associate editor of the Research Topic. All authors contributed to the article and approved the submitted version.

## Funding

JC is supported by Emerson Collective, NIH/NIAID (R21 AI155990), the Alvin J. Siteman Cancer Center through The Foundation for Barnes-Jewish Hospital, and the Alvin J. Siteman Cancer Center through The Foundation for Barnes-Jewish Hospital and the National Cancer Institute (P30 CA091842).

## Conflict of Interest

JC received an honorarium from Incyte Corporation and research funding from Mallinckrodt Pharmaceuticals.

The remaining author declares that the research was conducted in the absence of any commercial or financial relationships that could be construed as a potential conflict of interest.

## Publisher’s Note

All claims expressed in this article are solely those of the authors and do not necessarily represent those of their affiliated organizations, or those of the publisher, the editors and the reviewers. Any product that may be evaluated in this article, or claim that may be made by its manufacturer, is not guaranteed or endorsed by the publisher.

## Author Disclaimer

The content is solely the responsibility of the authors and does not necessarily represent the official views of the National Institutes of Health.

